# Validity and reliability of Turkish pregnant women's preferences for mode of delivery questionnaire

**DOI:** 10.1590/1806-9282.20231539

**Published:** 2024-05-27

**Authors:** Nurdan Kaya Yilmaz, Funda Evcili

**Affiliations:** 1 Ondokuz Mayıs University, Faculty of Health Sciences, Department of Midwifery – Samsun, Turkey.; 2 Sivas Cumhuriyet University, Vocational School of Health Care Services, Child Care and Youth Services – Sivas, Turkey.

**Keywords:** Delivery, Preferences, Questionnaire, Reliability, Validity

## Abstract

**OBJECTIVE:**

The aim of this study was to determine whether Pregnant Women's Preferences for Mode of Delivery Questionnaire, created by Zamani-Alavijeh et al., is a valid and reliable measurement tool for Turkish pregnant women.

**METHODS:**

This study has a methodological research design and was conducted with 139 pregnant women who were randomly selected from those aged 18–35 years, who applied to obstetric clinic,.who had no previous prenatal losses and no systemic diseases, and who had conceived naturally. The data for this study were collected with the Personal Information Form and the Pregnant Women's Preferences for Mode of Delivery Questionnaire. To test the reliability and validity of Pregnant Women's Preferences for Mode of Delivery Questionnaire, Cronbach's α, split-half method, item analysis, Kendall's coefficient of agreement (W), explanatory factor analysis, and confirmatory factor were used.

**RESULTS:**

The study found that Cronbach's α was 0.94, the Spearman–Brown reliability coefficient was 0.883, and the Guttman split-half was 0.880. Explanatory factor analysis revealed an 18-item structure with three factors having an eigenvalue exceeding 1, explaining 67.593% of the total variability, and factor loading between 0.40 and 0.64.

**CONCLUSION:**

Based on the scientific recommendations, the Turkish version of the Pregnant Women's Preferences for Mode of Delivery Questionnaire has adequate psychometric properties.

## INTRODUCTION

Delivery preference can be made when a woman voluntarily chooses between vaginal or cesarean delivery based on her knowledge, beliefs, and attitudes^1,2^. Vaginal birth is natural, normal, and suitable for female physiology^3^, while cesarean is an obstetric surgical method preferred in cases where vaginal delivery cannot be applied because of some maternal or fetal reasons, or when it is quite risky for the mother and the fetus. Cesarean rate has been increasing rapidly all over the world, and the current cesarean rate is 21.1%, which is expected to increase to 28.5% by 2030^4^.

During the last trimester, the healthcare team evaluates medical indications, discusses them with the mother and family, and decides on the mode of delivery. However, most women decide on the mode of delivery under the influence of social, psychological, and environmental factors, aside from medical indications^1^. Women's choice of elective cesarean is affected by their families, friends, media, hospital, previous birth experiences, and healthcare staff^5-8^. Cesarean can be lifesaving when necessary, but non-indicative and unnecessary cesarean has negative outcomes in terms of the health of the mother and the fetus/newborn^9,10^.

No questionnaire was found in the literature review to evaluate the birth method preferences of pregnant women in Turkey. Therefore, a measurement tool that can be applied in a short time by healthcare staff working in the perinatal field, can be easily interpreted, and will determine the factors affecting women's birth method preference is extremely necessary. Determining all the factors that affect the birth preferences of women, having the right information about birth patterns, and developing the appropriate intervention may be beneficial in decreasing cesarean section rates and increasing normal birth preferences. This study aimed to determine the reliability and validity of PPMDQ, which has not been used yet in pregnant women in Turkey.

## METHODS

### Study design and participants

This study was conducted using a methodological design in an obstetric clinic of a tertiary hospital between September 2020 and June 2021. It recommends that the number of samples must be between 5 and 10 times the number of items^11^. The study was conducted with a total of 139 pregnant women selected randomly from those who aged 18–35 years, who had no previous prenatal losses and no systemic diseases, and who had conceived naturally.

### Language adaptation protocol

In questionnaire adaptation studies, when the questionnaires are translated, the steps of "translation into the target language" and "translation back into the original language" are often followed. There must be compatibility between the original questionnaire and its translation, and the items are equivalent to each other^12^. First, the original questionnaire was translated from English into Turkish by three experts to ensure language and content validity. Second, the translated text was then translated back into the original language. Then, the translated form and the original questionnaire were sent to 10 different experts to select the most appropriate translation. These experts were asked to evaluate the compatibility of the translation items with the original items.

### Data collection instruments

The personal information form was used to collect the participants’ characteristics. The original questionnaire developed by Zamani-Alavijeh et al.^13^ consists of 21 items and 7 sub-dimensions. The questionnaire is in the 5-point Likert style (1= I Totally Disagree and 5=I Totally Agree). The Cronbach's α of the original questionnaire was determined to be 0.747^13^.

### Ethics statement

First, permission was obtained from the questionnaire developer. Second, this study was performed in accordance with the Helsinki Declaration and has been approved by the Ondokuz Mayıs University Clinical Research Ethics Committee (on February 13,.2019, with number B.30.2.ODM.0.20.08/48-184). All participants’ written consents were obtained.

### Statistical analysis

The data were transferred to the computer with the LISREL 8.54 and SPSS 22.0 package programs, and psychometric analyses were conducted. In this study, Cronbach's α, split-half method, and item analysis were used to test the reliability of PPMDQ. Kendall's coefficient of agreement (W) was calculated to determine whether the questionnaire's content was valid. The explanatory factor analysis was applied to test the construct validity of the questionnaire, and the confirmatory factor analysis was used to examine the relations between the questionnaire factors.

## RESULTS

The study found that the mean age of pregnant women was 28±4.17, with 93.5% living in nuclear families and 3.6% having low incomes. Women's gestational age was 24.64±10.42, with 47.5% in the third trimester, 63.3% preferred to have a vaginal delivery, 31.1% had a history of planned cesarean, and 71.2% received information about delivery.

### Reliability analysis

First, the study evaluated the item total score correlations of the 21-item questionnaire and three items that had a correlation coefficient below r=0.30 were removed from the questionnaire. After analysis, the number of items in the questionnaire decreased to 18. The remaining items had item total score correlation coefficients varying between 0.34 and 0.65, and the questionnaire items were adequate to represent the questionnaire (Table 1). After the item analysis, it was found that the Cronbach's α was 0.94, indicating high reliability. The Spearman–Brown and Guttmann split-half reliability coefficients obtained with the split-half method of the questionnaire were examined. It was found that the internal consistency coefficient of PPMDQ was Spearman–Brown (0.883) and Guttmann split-half (0.880).

**Table 1:** Distribution of Pregnant Women's Preferences for Mode of Delivery Questionnaire outline according to item total point correlation (n=139).

Items	Mean	Standard deviation	Corrected ttem total correlation	Cronbach's α if item deleted
i1	4.78	0.71	0.56	0.93
i2	4.85	0.50	0.74	0.93
i3	4.77	0.61	0.68	0.93
i4	4.71	0.71	0.68	0.93
i5	4.65	0.83	0.70	0.94
i6	4.66	0.83	0.70	0.93
i7	4.67	0.82	0.73	0.93
i8	4.67	0.71	0.69	0.93
i9	4.74	0.74	0.78	0.93
i10	4.71	0.76	0.70	0.93
i11	4.58	0.94	0.61	0.94
i12	4.65	0.74	0.67	0.94
i13	4.66	0.81	0.65	0.93
i14	4.69	0.75	0.75	0.93
i15	4.76	0.65	0.62	0.93
i16	4.64	0.86	0.62	0.94
i20	4.71	0.73	0.64	0.93
i21	4.65	0.72	0.64	0.94

PPMDQ: Pregnant Women's Preferences for Mode of Delivery Questionnaire.

### Validity analysis

The Kaiser–Meyer–Olkin (KMO) test (0.881) and Bartlett's test (^2^= 1894.713, SD=153, p=0.000) were found to be significant for PPMDQ, indicating that the data were suitable for factor analysis. The Kendall's W test was used to determine whether PPMDQ is valid in terms of content, and it was found that there were no statistical differences between expert opinions (Kendall's W: 0.176; p: 0.240>0.05).

The explanatory factor analysis (EFA) was made to test the construct validity and to determine the factors of the questionnaire. After the varimax factor rotation, a three-factor structure that had an eigenvalue above 1 and a factor load above 0.64 emerged, explaining 67.593% of the variance (factor 1: 52.370, factor 2: 8.262%, and factor 3: 6.961). The eigenvalues of the factors were found to be factor 1: 9.427, factor 2: 1.487, and factor 3: 1.253. After factor rotation, it was determined that 10 items were under the 1st factor, 5 items under the 2nd factor, and 3 items under the 3rd factor. Although the original questionnaire consisted of seven factors, a three-factor structure emerged in the Turkish validity and reliability study. These factors were named "belief," "self-efficacy," and "preferences" (Table 2).

**Table 2:** The results of explanatory factor analysis of Pregnant Women's Preferences for Mode of Delivery Questionnaire (n=139).

Factor	Item	Factor values	Eigenvalue	Variance
Factor 1. Belief	i6	0.583	9.427	52.370
	i7	0.442		
	i8	0.400		
	i9	0.563		
	i10	0.609		
	i11	0.817		
	i12	0.771		
	i13	0.791		
	i14	0.586		
	i15	0.799		
Factor 2. Self-efficacy	i1	0.790	1.487	8.262
	i2	0.585		
	i3	0.420		
	i4	0.697		
	i5	0.594		
Factor 3. Preferences	i16	0.841	1.253	6.961
	i20	0.758		
	i21	0.700		

PPMDQ: Pregnant Women's Preferences for Mode of Delivery Questionnaire.

Based on the EFA, the questionnaire that had a three-factor structure was tested with the confirmatory factor analysis (CFA). In the study, ^2^/SD=3.00, and the data fit of the model was found to be adequate. It was found that there was an agreement between the model and the observed data in terms of goodness-of-fit index values, and the validity and reliability study of the questionnaire for Turkish showed an acceptable level of fit (Table 3).

**Table 3:** Fit index values of the scale and standard fit index value ranges*.

Compliance measures	Standard fit index values	Acceptable fit index values	Scale's fit index values
RMSEA	0≤RMSEA≤0.05	0.05<RMSEA≤0.08	0.074
SRMR	0≤SRMR≤0.05	0.05<SRMR≤0.10	0.94
NFI	0.95≤NFI≤1.00	0.90≤NFI<0.95	0.93
CFI	0.97≤CFI≤1.00	0.95≤CFI<0.97	0.95
AGFI	0.90≤AGFI≤1.00	0.85≤AGFI<0.90	0.87
GFI	0.95≤AGFI≤1.00	0.90≤AGFI<0.95	0.91
x2/df	0≤x2/df≤2	2<x2/df≤5	396.01/132=3.00

* Standard fit index value^17^.

## DISCUSSION

The item total score correlation is used to determine the relationship between the scores obtained from individual test items and the total test score^14^. There are various evaluations for the lower correlation coefficient (r) limit. According to Buyukozturk^15^, the item total score correlation must be positive and greater than 0.30^15^. In the present study, three items that had a correlation coefficient below r=0.30 were removed from the questionnaire, and the 18 items with a correlation coefficient above r=0.30 were retained. The reliability criterion, also known as Cronbach's α, is used to evaluate the internal consistency of a Likert-type questionnaire. A Cronbach's α below 0.40 shows that the questionnaire is not "reliable," and if it is between 0.80 and 1.00, it shows that the questionnaire is "highly reliable"^12,14^. In the present study, the Cronbach's α of the 18-item questionnaire was calculated to be 0.94, and it was decided that the internal consistency of the questionnaire was highly reliable.

Another method employed to test the reliability of a questionnaire is the split-half method, which is the most widely used method for estimating test reliability^16^. The Spearman–Brown and Guttmann split-half reliability coefficients were examined in this study, and the internal consistency coefficient was found to be 0.883 and 0.880, respectively.

To test the validity of the questionnaire, the Kendall's W test and explanatory and confirmatory factor analyses were used. The Kendall's W goodness-of-fit test was used to determine the content validity of a questionnaire. It is aimed at determining whether there is agreement between expert opinions^15^. No significant differences were detected in the study in terms of expert opinions.

In this study, it was determined that although the original questionnaire had a seven-factor structure, the adapted questionnaire had a three-factor structure. When the questionnaire items were determined with the EFA, attention was paid to the fact that the eigenvalues of the items were 1, the load values were at least 0.30, the items were included in one single factor, and there was at least 0.10 difference between two factors^15^. The rotation of the factor load matrix helps find a more interpretable factor structure. The most commonly used technique in the rotation is the varimax, in which a rotation can be made with fewer variables so that the factor variances are maximized^14,15^. In this study, the "varimax method" was used as the factor rotation method. After varimax rotation, a three-factor structure that had an eigenvalue above 1 and a factor load above 0.64 emerged, explaining 67.593% of the variance.

The CFA was used to evaluate the accuracy of a structure determined by EFA. Goodness-of-fit tests are the steps at which the decision to accept or reject the model is made^14^. According to the literature data^17^, the standard fit index values show acceptable and good fit, and the fit index values of the questionnaire are given in Table 3. With the CFA, the fit index values of the questionnaire were found to be within the "acceptable" range. Depending on the degree of freedom, the low chi square value (^2^/SD) of 5 or fewer showed that the data fit of the proposed type is adequate^14^. In our study, ^2^/SD=3.00, and the model's data fit was found to be adequate.

This study has some limitations. First, the test–retest reliability analysis of the scale could not be performed due to the COVID-19 pandemic conditions. Second, the results are sample-specific. The research was carried out in a city. Therefore, researchers should also validate the PPMDQ in rural parts of Turkey. The PPMDQ is understandable and appropriate to the Turkish cultural context and can be reliable and valid for Turkish pregnant women.

## CONCLUSION

The Turkish version of the PPMDQ has adequate psychometric properties according to the best scientific recommendations. It was determined in our study that the data fit of PPMDQ according to fit index values was adequate, and the questionnaire could be used to determine women's types of delivery preferences. The questionnaire has 18 items and 3 sub-dimensions. The lowest score that can be obtained from the questionnaire is 18, and the highest score is 90. The questionnaire has no cutoff value. It is accepted that women prefer the normal delivery method as the score obtained from the questionnaire decreases, and the cesarean section method more strongly as the score increases. The questionnaire can be used by healthcare staff to evaluate women's beliefs about birth patterns, self-efficacy perceptions, and preferences for delivery methods, and to structure the contents of the training programs.

## LIMITATIONS

Owing to its single-centered nature, the results cannot be generalized to all pregnant women.
